# Unexpected endometrial malacoplakia related to abortion and placental rests retention: a case report

**DOI:** 10.1186/s13000-020-01014-x

**Published:** 2020-07-18

**Authors:** Antonio d’Amati, Emilio Bellitti, Leonardo Resta

**Affiliations:** Section of Pathology, D.E.T.O. (Department of Emergency and Organ Transplantation), University of Bari, Piazza Giulio Cesare 11, 70124 Bari, BA Italy

**Keywords:** Pathology, Malacoplakia, Endometrial diseases, Abortion, Case report

## Abstract

**Background:**

Malacoplakia is a rare chronic inflammatory disease. The name derives from the Greek “μαλακός” meaning “*soft*” and “πλάξ” meaning “*plaque*”, describing its usual macroscopic presentation as a friable yellow soft plaque. It was first described by von Hansemann in 1901 and by Michaelis and Gutmann in 1902. The urinary system is the most commonly involved site. Female genital tract involvement is extremely rare. Treatment is prevalently based on antibiotics with surgical intervention sometimes necessary. Prognosis is usually good, but relapse may frequently occur.

**Case presentation:**

This report illustrates the first case of endometrial malacoplakia in a 40 years-old patient who received endometrial curettage due to the retention of placental rests following an abortion. After conspicuous vaginal sero-hematic secretions, the patient received a further curettage. The histological examination did not show any retention of chorionic rests, but an endometrial and myometrial infiltration of histiocytes with large granular cytoplasm within a chronic inflammatory background. Immunoreactivity for CK-pool was negative, while CD68 immunostaining was strongly positive.

**Conclusions:**

Malacoplakia of endometrium is an extremely rare condition, with few cases reported in the whole international literature. In this paper, we present the first case associated to an abortion followed by endometrial curettage procedures. This rare disease should always be attentively examined, considering, among differential diagnoses, uterine neoplasms or physiological conditions such as cumulus of foamy macrophages in the endometrium.

## Background

Malacoplakia is a rare chronic inflammatory disease, with a mean age at diagnosis of 50 years old and a female to male ratio of 4:1. It occurs more frequently in patients with immunodeficiency conditions. The disease was first described by von Hansemann in 1901 and by Michaelis and Gutmann in 1902. The name malacoplakia derives from the Greek words “μαλακός”, which means soft, and “πλάξ”, meaning plaque, and is related to its macroscopic appearance: soft and friable yellow plaques or, more rarely, nodules or masses. Histologically, it is characterized by a mixed inflammatory infiltrate, composed of lymphocytes, plasma cells, neutrophils and aggregates of hystiocytes with abundant granular eosinophilic cytoplasm, known as von Hansemann cells. The genitourinary tract is the most common involved site, followed by the gastrointestinal tract, but any site of the human body may be affected. The female genital tract is a very rare localization, with only few cases reported in literature, involving vagina, uterus, fallopian tubes and ovary. The most common presentation of endometrial malacoplakia is post-menopausal bleeding. We report herein the first case of endometrial malacoplakia associated to an abortion followed by uterine curettage procedures.

## Case presentation

**Fig. 1:**
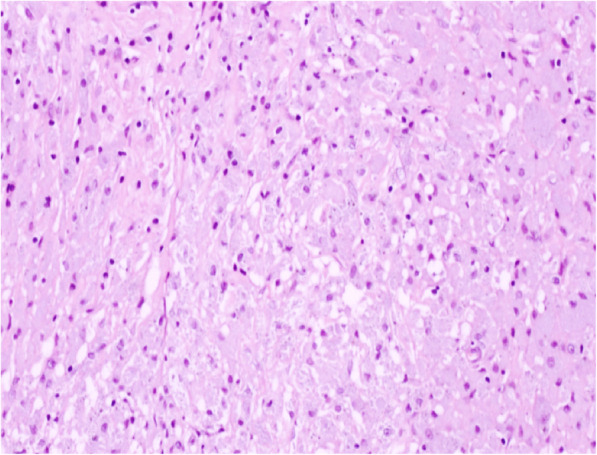
Chronic inflammatory infiltrate, mostly composed of histiocytes with abundant granular cytoplasm and basophilic bodies. (H&E stain, 100x)

**Fig. 2:**
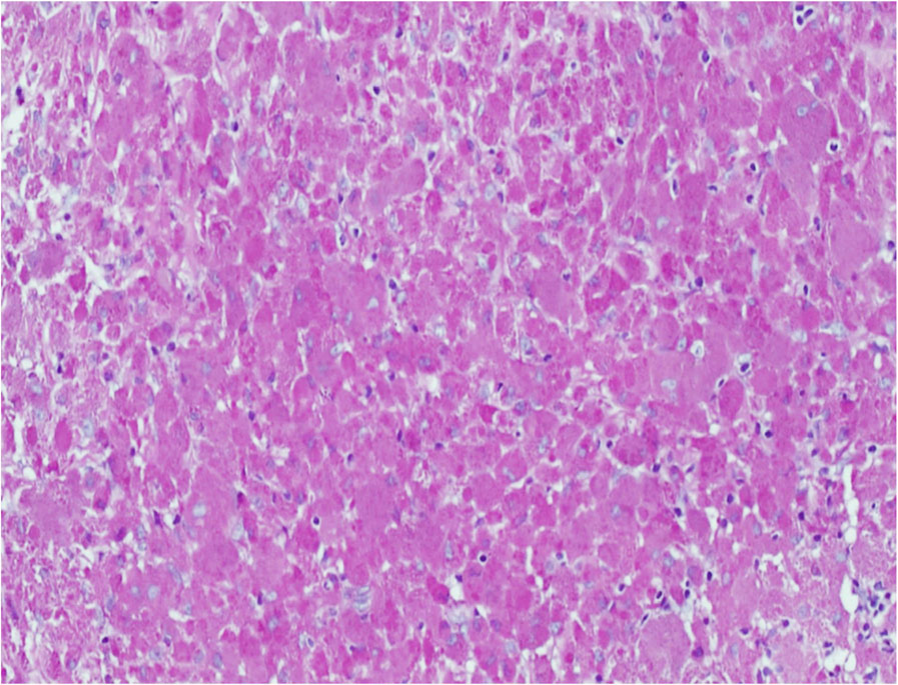
Diffuse cytoplasmic staining in histiocytic elements. (Periodic acid-Schiff stain, 100x)

**Fig. 3:**
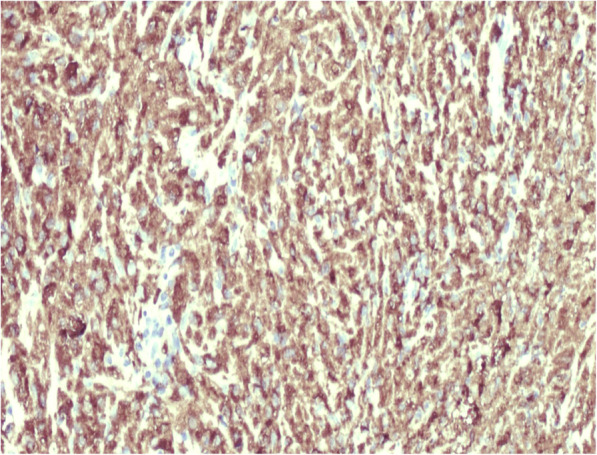
Strong cytoplasmic positivity for CD68, highlighting several histiocytes. (100x)

**Fig. 4:**
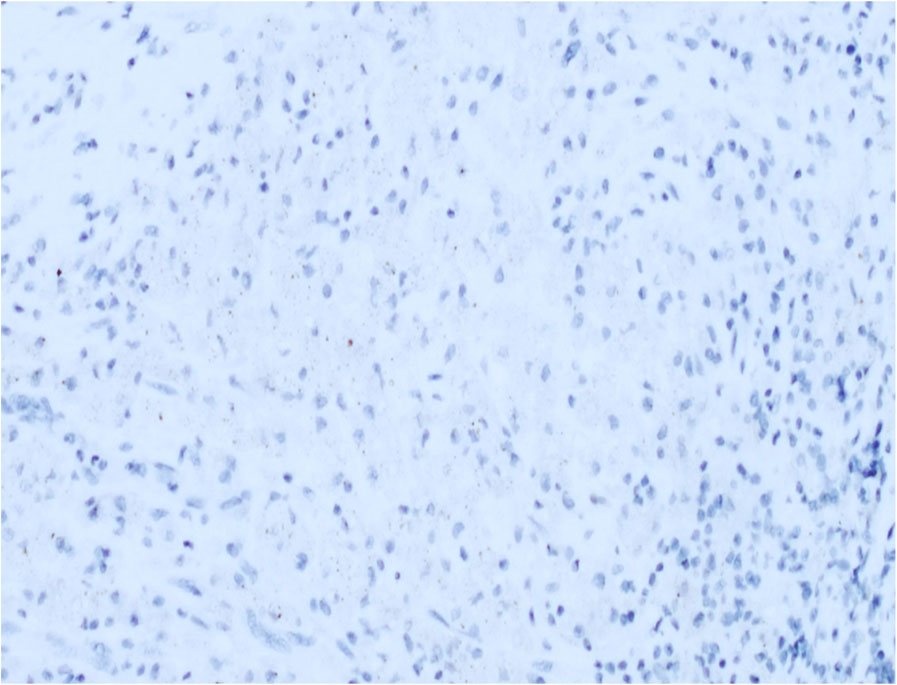
Negative immunoreaction for CK-pool. (100x)

A 40-year-old female patient was admitted to our hospital for conspicuous vaginal sero-hematic secretions. Anamnestic information asserted that the patient previously received three endometrial curettage after a surgical abortion procedure. Histological examination after each endometrial curettage showed regression of chorionic villi and decidualization of the endometrium and the diagnosis of placental rests retention was made. After being admitted for conspicuous vaginal sero-hematic secretions, the patient received a further curettage in order to assess the possible retention of placental rests. The histological evaluation did not show any retention of chorionic rests, but demonstrated an endometrial and myometrial diffuse chronic inflammatory infiltration, with an extensive background of hyaline stroma. The inflammatory infiltrate showed a variegate composition, being made of lymphocytes, plasma cells and occasional polymorphonuclear leukocytes, but the majority of the cellular elements was represented by histiocytes with abundant granular cytoplasm and basophilic inclusions, morphologically relatable to the so called von Hansemann cells (Fig. 1). Periodic acid-schiff (PAS) reaction highlighted a diffuse cytoplasmic staining in von Hansemann cells (Fig. 2). The immunohistochemical analysis revealed that those elements were strongly positive for CD68 (Fig. 3) and negative for CK-pool (Fig. 4). On the basis of morphological, histochemical and immunophenotypical evidence, the histological diagnosis of malacoplakia was made, excluding uterine neoplasms and other differential diagnoses. In the following months, due to relapse of clinical manifestations, nonetheless administration of antibiotic drugs, the patient underwent two further uterine curettage, with the diagnosis of recurrent endometrial malacoplakia.

## Discussion and conclusions

Endometrial malacoplakia is an extremely rare condition, as demonstrated by the paucity of cases reported in the international literature. The etiopathogenesis of the disease is far from being completely understood, but recent evidence agrees with the presence of defective macrophage phagolysosomal activity [1]. Several studies reported a correlation between malacoplakia and immunodeficiency conditions, such as primary immunodeficiency, steroid administration, AIDS and diabetes [2]. The defect in the phagolysosomal activity is believed to be a consequence of immunodeficiency, leading to incomplete bacterial digestion and to Michaelis-Gutmann bodies formation. These bodies are considered as a pathognomonic microscopical sign of malacoplakia, but they are not strictly necessary for the diagnosis, as they may not be seen in early stages of the disease. The case presented in this report developed endometrial malacoplakia after an abortion and subsequent uterine curettage due to retention of placental rests. On the basis of the clinical history, we might suppose a bacterial contamination leading to the development of the disease, but in the absence of a known condition of immunodeficiency. Moreover, due to placental rests retention, we might hypothesize a role played by trophoblast. In fact, cytokines released by trophoblast cells have been reported to be involved, among various functions, in immunoregulation and maternal-fetal tolerance during pregnancy [3, 4]. The clinical presentation of endometrial malacoplakia is characterized by post-menopausal bleeding, abnormal uterine bleeding in a menstruating woman or suspicious uterine mass [5–7]. Our patient presented with abundant vaginal sero-hematic secretions and received a further curettage, in order to establish a possible placental rests retention, but the histological examination revealed the microscopical features of endometrial malacoplakia and the absence of chorionic rests. Due to its clinical presentation and endoscopical appearance, uterine malacoplakia requires a careful differential diagnosis, in order to rule out other inflammatory processes and uterine neoplasms. The therapeutic approach to malacoplakia is based on surgical and medical options, but there are no established guidelines. Surgical intervention varies in accordance to site and extension of the disease. Antibiotic therapy is prevalently based on drugs capable of reaching high concentrations inside macrophages, such as quinolones or trimethoprim-sulfamethoxazole [8, 9]. Currently, antibiotics, combined with surgery, provide the best therapeutic protocol. A novel protocol, including antibiotics, bethanechol and ascorbic acid, has been recently used in the treatment of cerebral malacoplakia [10]. In the case herein reported the patient was treated with uterine curettage, combined with quinolones antibiotic therapy, obtaining a rapid clinical improvement, with satisfactory disappearance of symptoms, as also reported by the patient. Even though management of this rare condition is very challenging, the prognosis is commonly good. Nevertheless, recurrence, as in the case we reported herein, and complications may frequently occur throughout the years.

## Data Availability

Not applicable.
